# Dance as mindful movement: a perspective from motor learning and predictive coding

**DOI:** 10.1186/s12868-024-00894-9

**Published:** 2024-11-06

**Authors:** W. Tecumseh Fitch, Rebecca Barnstaple

**Affiliations:** 1 Dept. of Behavioral and Cognitive Biology, University of Vienna, Vienna, Austria; 2 Theatre Studies, University of Guelph, Guelph, ON Canada

**Keywords:** Dance, Motor control, Predictive processing, Automatization, Consciousness

## Abstract

Defining “dance” is challenging, because many distinct classes of human movement may be considered dance in a broad sense. Although the most obvious category is rhythmic dancing to a musical beat, other categories of expressive movement such as dance improvisation, pantomime, tai chi, or Japanese *butoh* suggest that a more inclusive conception of human dance is needed. Here we propose that a specific type of conscious awareness plays an overarching role in most forms of expressive movement and can be used to define dance (in the broad sense). We can briefly summarize this broader notion of dance as “mindful movement.” However, to make this conception explicit and testable, we need an empirically verifiable characterization of “mindful movement.” We propose such a characterization in terms of predictive coding and procedural learning theory: mindful movement involves a “suspension” of automatization. When first learning a new motor skill, we are highly conscious of our movements, and this is reflected in neural activation patterns. As skill increases, automatization and overlearning occurs, involving a progressive suppression of conscious awareness. Overlearned, habitual movement patterns become mostly unconscious, entering consciousness only when mistakes or surprising outcomes occur. In mindful movement, this automatization process is essentially inverted or suspended, reactivating previously unconscious details of movement in the conscious workspace, and crucially enabling a renewed aesthetic attention to such details. This wider perspective on dance has important implications for potential animal analogs of human dance and leads to multiple lines of experimental exploration.

## Background

A central problem confronting an empirical exploration of the biological basis of human dance is definitional: what counts as “dance”? A definition, even if provisional, is important to understanding both the neural basis of dance, and in seeking possible dance analogs in nonhuman animal movement. Depending on the conception of dance chosen, different categories of human and animal displays may be more (or less) relevant to understanding the biological roots of dance.

The most obvious category of dance involves rhythmic bodily movement entrained to a beat provided by musical accompaniment (“dancing to music”). For many scholars, this narrow but well-defined class of movement-to-music is prototypical of dance (e.g., in the comprehensive review of [1]). Another narrow, often overlapping, interpretation of dance involves two or more synchronized individuals (“dancing with others”). Recognized social dance forms, from waltz to salsa to kizomba, fall under both of these conceptions, as do performance practices such as traditional ballet. Most folk and street dances, many ritual or ceremonial dances, and a host of other cross-culturally widespread dance phenomena are naturally included within these narrow definitions of “dance” as rhythmic movement to music and/or with others. We fully applaud their study, and we have little doubt that music and dance have been tightly linked throughout human evolution [2, 3].

However, narrow conceptions of dance cause two major difficulties for inquiries into the biological basis of dance. First, many expressive and aesthetically powerful forms of human movement are excluded by a narrow, musical-beat-based, conception of dance, which neglects other aspects of engagement with movement and cultural knowledge that are central to the broader phenomenon of dance. Movement traditions like tai-chi or yoga, Western performance styles such as mime, physical theatre, performance art, much modern dance, and “mummer plays” all often lack music, and may be performed alone. It excludes a host of non-Western cultural traditions such as Indian *kathakali*, Hawai’ian *hula*, or Japanese *butoh*. Sign language poetry is another form of expressive movement [4] that by nature involves no sound [5]. Finally, codified movement systems like gymnastics or figure skating are typically classed as “sport” but are close cousins to dance. All of these practices are complex, learned, and involve expressive movement sequences that provoke powerful aesthetic reactions from both practitioners and audiences, but do not necessarily involve entrainment to musical or other rhythmic accompaniment. We argue that these culturally codified forms of movement are potentially relevant to developing a deeper understanding of the biological roots of dance, and that their exclusion from consideration as “human dance” risks causing both theoretical and practical/empirical problems.

**Fig. 1:**
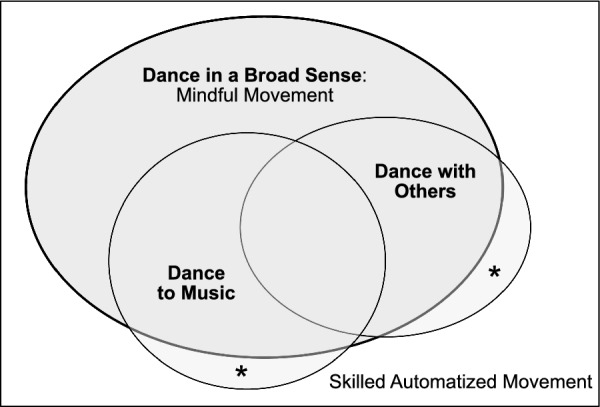
**Dance in a Broad Sense**—The outer rectangle includes any skilled movement. Subsets of this wide field include “mindful movement”—our proposed definition for “dance in a broad sense” or DBS. Mostly overlapping with DBS are narrower conceptions of dance, such as dancing to music and/or dancing with others—which themselves often overlap. However, it is possible to perform dance to music in a skilled but “mechanical” manner: “going through the motions” with little attention to the dance movements, and thus without being truly immersed and mindful. By our definition, such instances would be excluded from DBS, and are marked with asterisks in the figure

Equally relevant, when adopting a narrow conception of dance as movement to music, clear analogs or precursors are quite rare among nonhuman animals. Neither chimpanzees, nor most other primates or mammals, flexibly entrain their movements to an external beat [6, 7], and directly comparable capacities are rare in the animal kingdom. The best documented examples of “dancing animals” come from parrots: Multiple parrot species are capable of extracting a regular beat from auditory stimuli and flexibly entraining their movements to this beat [6, 8–11]. Parrots are very distant relatives of humans, and most other birds and mammals lack this capability; parrot “dancing” thus represents a convergently evolved ability. Another example comes from a sea lion who learned to bob its head to a musical beat [12]. Various other forms of animal entrainment are sometimes considered relevant to human dancing [7, 13, 14] but the degree to which they involve flexible synchronization like that seen in human “dance to music” is less clear. We suggest here that a broadened conception of dance, incorporating a wider range of motor displays observed in a larger variety of species, may prove more fertile for comparative work (Fig. 1).

For these reasons, the aim of this paper is to introduce a broader notion of what counts as “dancing”: dance in the broad sense (DBS). Simply put, we conceive of DBS as “mindful movement”. We offer an empirically testable characterization of this use of “mindfulness”: mindful movement involves heightened attention to the dynamics and expressive potential of movement, particularly towards details that are typically ignored during ordinary automatized action. This awareness of dynamic expressive possibilities is often coupled with an expressive goal, and involves refining the movement toward this goal. Motor activity in this characterization incorporates both the sensory effects of movements, and how one is positioned within and interacts with the environment (often including other dancers).

We argue that this broader definition of “dance” incorporates many expressive forms of human movement excluded by narrower definitions and provides new routes to understanding the evolution of dance by greatly broadening the comparative database to include a more widespread set of animal display behaviors beyond “dance to an auditory beat”. We will suggest here that this broader conception of dance has important testable implications for empirical research in aesthetics, the neuroscience of dance, and the use of dance in clinical settings (see “Neural correlates” below).

The rest of the paper will be devoted to clarifying our conception of DBS and exploring some of the consequences of adopting it.

## Defining “Mindful Movement” in a motor learning context

The term “mindfulness” has multiple distinct interpretations, ranging from effortful, focused attention on one’s internal state in mindfulness meditation, to enhanced attention to goals and outcomes in some mindful movement paradigms (for some key distinctions see Clark 2015). Without further clarification, the term “mindful” thus seems too ambiguous to guide empirical research. An initial rough definition of mindfulness is “the awareness that emerges through paying attention on purpose, in the present moment, and non-judgmentally to the unfolding of experience” [15, p. 145]. Note that by this definition, mindfulness emerges intentionally, by consciously directing attention towards the finer details of experience. We build upon this key notion by suggesting more specifically that “mindful movement” involves the conscious allocation of attentional resources to aspects of previously automatized motor control that, under ordinary circumstances, remain unconscious. The term “mindful movement” has been used in the field of “somatic education”, a constellation of intentional movement practices involving mindfulness and conscious action and that explicitly includes non-dance movement [16]. However, our use of the term here in a dance science context has only partial overlap with this previous usage.

We focus on mindful *movement* here both because our focus is dance, and because the domain of movement offers several specific empirical advantages. Movement in general provides rich sensory feedback, inviting attention with little effort or top-down control [cf. 17]. Motor control and motor learning are paradigmatic domains for predictive coding frameworks [18–22], because sensorimotor feedback is especially salient when outcomes are unexpected (e.g. when a grasped object is heavier or lighter than expected). In particular, motor learning theory posits prominent attention to details of sensory feedback during early learning stages, but a gradual suppression of awareness as the behavior becomes overlearned or “automatized” [22–24]. Our notion of “mindful movement” builds upon these notions, and thus focuses on skilled, habitual and overlearned movement patterns, ranging from basic dance steps, techniques like *en pointe* dancing in ballet, or sophisticated higher-order assemblages of such patterns. This focus provides a well-defined and replicable context, well-suited to empirical investigation and experimental data collection.

## Attention and automatization in motor skill learning

Standard cognitive models of attention and memory assume that there are sharp limits on attention (we can’t pay attention to everything) and that the contents of working memory are equally tightly constrained [25, 26]. Furthermore, the vast majority of neural processing happening at any moment in our nervous systems is unconscious. In some cases this is inevitable, because we could not in principle attain conscious access to the processing involved (e.g., the neural processing carried out by our digestive systems, low-level spinal circuitry, or the retina). However, in other cases, access to processing is available in principle, but not typical in practice when executing overlearned activities. Motor skill learning (“procedural learning”) provides a paradigm case [22–24]: Skilled, overlearned behaviors become “automatized” during skill learning, meaning that they are initially effortful and fully conscious, but we can eventually deploy the learned behavior with minimal conscious attention. Riding a bike, driving a car, typing familiar words or playing well-mastered musical pieces are relevant examples.

At first, procedural learning of motor skills involves considerable attention and conscious cognitive effort. However, as learning progresses, the attentional demands of the learned action steadily decrease, until with practice, the action becomes “overlearned,” akin to a reflex, happening with little need for conscious attention. The initial “cognitive” stage in early motor learning clearly demonstrates that details of overlearned actions are *potentially* available for consciousness. However, once learning and automatization have occurred, overlearned actions require (and typically receive) minimal conscious attention: they are results-oriented, focused on achieving specific goals efficiently. Automatization is highly adaptive: it frees attentional resources for other ongoing activities, permitting engagement with novel more challenging tasks. For complex action sequences, automatization of subsequences also allows attentional resources to focus on more sophisticated action sequences, hierarchically building upon previously automatized actions.

To illustrate, when learning a new partner dance such as waltz or salsa, linking the novel pattern of foot movements to the musical rhythm may initially be quite challenging, requiring full conscious attention. But with experience, these movements become automatized, freeing the dancer to acquire more complex actions that build upon (and require mastery of) the basic step, such as twirls, dips, or flourishes. These higher-order action complexes can themselves become automatized with time, and support sequences at still higher levels of complexity (e.g. a “dance routine”). For the experienced dancer, the result of the extended learning process involved in learning a new dance style is a hierarchically structured repertoire of potential motor actions, flexibly available for deployment with minimal cognitive effort. This “motor toolkit” allows skilled dancers to focus on subtle high-level issues such as style, improvisation, dynamic expression, and interaction with the partner, without conscious attention to the dance steps or finding the beat in the music.

Less rhythmically constrained dance forms still rely on conscious attention to refinement of skills such as expressing an experience or embodying a metaphorical image [27]. For example, contact improvisation relies on honing sensitivity to the effects of gravity—novice dancers may start with individual floor work before interacting with other dancers in more complex and risky explorations. Ravn and Høffding aptly describe improvisation in dance as a “a curiosity-driven urge to explore the body and our relations to others and involving different ways of using attention” [28, p. 518], consistent with our notion of mindfulness in DBS. A range of attentional strategies are available during skill learning, where internal versus external focus of attention is often distinguished [29]. Ballet dancers self-report having a mainly internal focus [30], and motor control theory suggests that differences in focus of attention act to allocate precision to either bodily movements or to goal attainment [31].

In either case, the ability to perform complex skills with less cognitive effort first requires many hours of consciously attending to and refining techniques related to the skills in question. In our usage, “mindful movement” implies a complex relationship between training, practice and intention. The performance of any skilled activity cannot be considered apart from the hours of training, practice, and “enskillment” [32] leading up to it. *Technique* can be considered as the application of specific and accumulated skills-based knowledge that evolves from episodes of *practice*, which deploy and (potentially, mindfully) extend that knowledge [33]. This is a useful distinction for our discussion of dance, as it applies to many variations in form; the *bananeira na cabeca* (the ‘Banana Tree’), a capoeira maneuver in which the entire weight of the body rests on the head, is offered by Downey as an example of an expert technique which “entails not so much the internalisation of a model movement as the realisation of a fundamental quality in the body—the extraordinary strength and resilience of the spine and head”, made possible only through extensive training and practice [32, p. 85].

Physiological alterations resulting from a gradual accumulation of automatized sub-skills have been referred to by Spatz as *sedimented agency*: the extent to which the the body is shaped through technique [33, p. 56]. Spatz further asserts that “we cannot assume (a) causal link between consciousness and agency…we must expand our idea of agency so that it extends beyond the conscious mind” (ibid., p. 55). Technical expertise, involving automatized movements resulting from hours or sometimes years of practice, clearly fits this description of sedimented agency. This model provides fertile theoretical grounds for applying empirical approaches in the investigation of mindful movement.

It might be tempting to expand our definition beyond previously automatized movements, and thus to include *all* mindful movement as dance. We resist this temptation for the following reasons. First, any movement by a novice learning a new task, and virtually all movements of young children, would have to be considered “dance” if we included ANY mindful movement as “dance in a broad sense.” In our opinion, such a definition would be overly inclusive and expand the category far beyond what the word “dance” connotes in English. Second, much of the methodological promise of our current definition would be lost in such a broad definition, since (as we argue in more detail below), the crucial tests to empirically characterize “mindfulness” will rely on the contrast between the movements of a novice learner, which are by default mindful, and those of the experienced actor, in which mindfulness is optional. For both of these reasons, we restrict our definition to skilled, previously automatized movements: dance in our broad sense involves “re-minding” automatized, and thus potentially robotic and inexpressive, actions.

We are now in a position to specify more precisely what we mean by “mindful movement.” During skilled motor activity, conscious cognition normally operates at a high level, focused on achieving goals and paying little attention to automatized details of movement and motor planning. Although lower-level sensorimotor feedback is *in principle* accessible to consciousness (as evidenced during early stages of motor learning), during automatized action we normally become conscious of this feedback only when predictions fail. Mindful movement involves consciously directing attention to these normally unconscious aspects of automatized action, thus “inverting” or temporarily suspending the suppression of awareness typical of automatization. This inversion, we predict, will be accompanied by a re-activation of previously suppressed activity in higher-level brain regions that are normally associated with initial skill learning.

## Neural correlates of “mindfulness” in dance

We have argued that it will be valuable in dance research to broaden the scope of inquiry to incorporate forms of movement that are “mindful” (involving heightened attention to refining dynamic and expressive aspects of movement), but not necessarily performed with music or other dancers. While we hope to have clearly defined this key basis for “dance in a broad sense” phenomenologically (from an internal, subjective viewpoint), dance science will need objective measures to unlock the full potential of this broadened scope. This will be particularly crucial for investigations of nonhuman animals, who cannot linguistically report their subjective degree of heightened motoric attention. To achieve this, we envision a research programme in which research on adult humans, exploiting the power of language for specific instructions and participant feedback, leads the way to the discovery of robust objective measures of mindfulness in movement. These measures could then be applied to nonhuman animals (or nonverbal humans, such as infants or patients).

Crucially, our perspective is dynamic and gradual: neural organisation is not “hard-wired” before or after practice. This dynamic conception of skill acquisition in mindful dance implies that experience, learning, and attention alter synaptic connections, reflected in structurally and functionally measurable brain changes. To clarify this key aspect of our proposal, we adopt a modern predictive processing perspective on cognition, which holds that minds are predictive systems. Specifically, at the neural level, computations mostly focus on error signals—situations when prediction fails—rather than faithfully transmitting bottom-up information [18, 19, 34]. In motor control, we constantly compute the expected outcomes and sensory consequences of voluntary actions (using “forward model” circuitry), and typically suppress sensory input compatible with these predictions [21, 22]. Further processing of well-predicted outcomes is unnecessary, and predictions that are fulfilled can thus be “subtracted” or “cancelled out” from higher-level cognitive computations. The brain thus conveys prediction *errors* to higher levels of processing, and not raw sensory details. Crucially, for automatized actions, *sensory feedback is highly salient only when predictions fail*—when expected outcomes do not materialize.

This provides a framework in which the degree of “mindfulness” in movement can be rendered empirically testable in motor learning paradigms. Starting with a particular overlearned action (perhaps learned in the course of earlier sessions), we can experimentally titrate attention to the movement. Attention can be increased by occasionally violating the predicted outcomes of the action (e.g. in perturbation experiments), or decreased by creating an attentional load for example by having participants engage in some unrelated task in an interference paradigm. Such manipulations allow us to distinguish between ordinary (unconscious) motor processing and conscious movement, and thus to extract neural signatures of conscious attention or “mindfulness”. In situations of mindful movement, when attention is paid to normally unconscious components of motor control and sensorimotor feedback, similar neural signatures should be observed even in the absence of prediction failure.

Specifically, during learning movement should be associated with greater activation of higher-order somatosensory, associative, prefrontal and premotor areas. As automatization occurs, these cortical activations will grow weaker, while subcortical motor activations (e.g. in basal ganglia and cerebellum) are expected to persist. During “mindful movement,” this automatization should be inverted, and the higher-order cortical activations in areas like SMA or somatosensory cortex are expected to return. Thus, at the simplest level, we suggest that the initial stages of skill learning are more mindful and thus involve considerable conscious attention and effort.

Some examples of proposed neural correlates for the processes of automatization in dance are provided by studies on expert dancers. For example, an fMRI (functional magnetic resonance imaging) study of professional ballerinas learning a new choreographed sequence [35] found that activations in cortical regions associated with motor learning and sequencing (particularly supplemental motor area—SMA) increased during the initial acquisition and refinement stages (7 weeks), but that this signal decreased significantly once the choreography was “overlearned” and regularly performed (34 weeks). Learning-based plasticity associated with skill acquisition has also been reported in relation to circus arts such as juggling [36].

While it is of course quite difficult to study actual dancing in an MRI scanner (or its magnetic analog MEG), empirical indications of increased higher-order activations are also detectable using wireless brain imaging caps that can be worn during free movement [37]. Several relevant studies are currently available. In an important recent example, using mobile brain/body imaging (MoBI) based on fNIRS (functional near-infrared spectroscopy), Ono and colleagues have analyzed brain activation in participants receiving video-game-based training on a dance routine [38, 39]. They documented a gradual decrease in top-down cortical activations, and a relative increase in bottom-up sensory input, as dance mastery progressed. We predict that, during mindful execution of such a learned routine, some components of these previously suppressed frontotemporal activations would be reactivated in direct proportion to self-reported mindfulness.

EEG (electroencephalography) provides another important MoBI methodology that, due to its higher temporal resolution, can provide richer dynamic information concerning attention to movement than fNIRS [40]. An early study using this approach showed that neural classifiers can predict, based on low-frequency delta band (0.2–4 Hz) EEG dynamics, categories of expressive movement evoked via verbal instruction in highly trained Laban movement practitioners [41]. Coupled with training studies, mobile EEG could provide a rich source of objective measures of DBS. In particular, we predict that analyses of cross-frequency coupling between low (delta) and higher (beta/gamma) EEG bands will increase during mindful versus automatized movement, and in general that associative cortical activations should be strongly and causally coupled to motor activations during mindful movement.

Using methods like these, our proposed definition of “mindful movement” provides a way to empirically evaluate and quantify the degree to which a reallocation of conscious attention to movement has occurred during learned action. Although controlled experiments varying subjectively perceived “mindfulness” of movement will be needed to provide more specific correlates than this, this research illustrates how neural investigation can be used to test predictions of our notion of “mindful movement.” Thus, while it is clearly too early to robustly specify the expected neural correlates of DBS, we see MoBI technologies as providing a feasible and promising research program for the future. Although initially deployed with adult human subjects using language, such technology will pave the way to comparative studies because the measures derived can then applied to other species.

## Conclusions: implications of a broader notion of dance

As we hope is clear, our notion of dance as mindful movement easily incorporates a wide range of human behaviors excluded by narrower “dance to music” or “dance with others” definitions (Fig. 1). Many performances explicitly labelled “modern dance,” along with mime, performance art, and Japanese *butoh*, eschew musical accompaniment but nonetheless are often considered forms of dance. Furthermore “sports” like figure skating, karate, and gymnastics may certainly qualify as dance by our broader definition. The definition offered here potentially views these and other classes of movement as “dance” *if* they are performed mindfully, with a clear and conscious attention to dynamic expressive details of the movement.

In an excellent investigation of the relevance of mindfulness to dance in our broadened definition, dance researcher Carolina Bergonzoni undertakes a practice of “dancing-a-walk” to explore the shift in experience that occurs when bringing intentionality to the functional/habitual movement of walking [42]. Her core argument is that walking can be transformed into dance by “a self-awareness of the body and its movement”. She writes: “When dancing-my-walk, I perceive a familiar shift of energy and intentions in my body that I recognize as a dance. I define walking as an unreflective, habitual movement that does not need focus or explicit attention; it flows by itself without being guided by reflective thoughts. On the other hand, to dance-a-walk is explicitly expressive even though the action (walking) itself is still unreflectively habitual. To dance-a-walk requires commitment, concentration, focus, and the explicit intention of transforming the action of walking into an expressive walk” [42, p. 34]. Following Bergonzoni, we would be prepared to suggest that any automatized complex action pattern (e.g. chopping carrots or canoeing through a swamp) *could* be “dance” when executed mindfully.

Bergonzoni also investigates whether this shift in internal perception or attitude is visible to outside observers. Accepting Bergonzoni’s argument that subjective mindfulness is often perceptible to observers, “mindfulness” could be quantified via third-party evaluations in psychological rating studies. Furthermore, we hypothesize that perceived mindfulness and expressivity is often more relevant to a viewer’s aesthetic judgements than, for example, whether the dancer is correctly implementing the right movement patterns at the right times (“going through the motions” without feeling). This is consistent with the intuition that music can be performed “with feeling” or “mechanically,” and can be explored using parallel psychological and psychophysiological measures (e.g. skin conductance or facial EMG in onlookers: [43]).

Our broader conception of dance potentially incorporates many forms of animal movement that may be relevant to the evolution of human dance and stylized movement. In particular, most vertebrate species employ species-typical motor displays during courtship and/or territorial defense, in which fine details of movement execution are central factors, critical to successful displays [e.g., 44, 45–48]. Such motor displays are often primarily visual, although potentially accompanied by sounds. However, very few are entrained to periodic sounds as would be required to be considered “dance” in a narrow sense. Examples include foot-waving frogs, who ostentatiously extend first one leg and then another to attract mates, “tap dancing” birds that perform a rapid and elaborate pattern of footfalls during displays, or males in numerous mammal species that perform impressive visual displays such as stiff walking with piloerection and upright horns or antlers during territorial encounters with other males [49–51]. In all of these cases, the displayer focuses on the movement as a goal in itself, and fine differences in execution, if perceived by mates or competitors, may determine the success or failure of the display. We suggest that such displays may fit our definition of “mindful movement,” and may thus be relevant to understanding the broader aesthetic aspects of human dance from both evolutionary and neural viewpoints.

Finally, note that mindfulness in our sense is a graded state: one can be more (or less) mindful (and probably never “fully” mindful). Our definition of mindfulness thus suggests a continuum whereby some motor performances might be more “dance-y” than others. At one end of this scale, truly automatized movements with no conscious attention would be excluded (walking or driving, or dancing the hokey-pokey or tango “without feeling,” see Fig. 1). We would also exclude attention to movement only when surprises or mistakes occur (despite potential activation of cortical areas by such prediction errors). But under ideal circumstances, in a highly mindful state, a skilled and expressive performer allocates nearly all conscious attention to performance details of the action itself. This intentional mindful state permits greater nuance, dynamic range, demonstration of proficiency, and heightened potential for expression: all core features of dance across artistic and cultural domains.

## Data Availability

No datasets were generated or analysed during the current study.
